# Identification of Comprehensive Geriatric Assessment Based Risk Factors for Malnutrition in Elderly Asian Cancer Patients

**DOI:** 10.1371/journal.pone.0156008

**Published:** 2016-05-27

**Authors:** Tira Tan, Whee Sze Ong, Tanujaa Rajasekaran, Khai Nee Koo, Li Li Chan, Donald Poon, Anupama Roy Chowdhury, Lalit Krishna, Ravindran Kanesvaran

**Affiliations:** 1 Division of Medical Oncology, National Cancer Centre Singapore, Singapore, Singapore; 2 Division of Clinical Trials and Epidemiological Sciences, National Cancer Centre Singapore, Singapore, Singapore; 3 Perdana University Graduate School of Medicine, Selangor, Malaysia; 4 Raffles Cancer Centre, Singapore, Singapore; 5 Duke-NUS Graduate Medical School, Singapore, Singapore; 6 Changi General Hospital, Singapore, Singapore; National Cancer Center, JAPAN

## Abstract

**Purpose:**

Elderly cancer patients are at increased risk for malnutrition. We aim to identify comprehensive geriatric assessment (CGA) based clinical factors associated with increased nutritional risk and develop a clinical scoring system to identify nutritional risk in elderly cancer patients.

**Patients and Methods:**

CGA data was collected from 249 Asian patients aged 70 years or older. Nutritional risk was assessed based on the Nutrition Screening Initiative (NSI) checklist. Univariate and multivariate logistic regression analyses were applied to assess the association between patient clinical factors together with domains within the CGA and moderate to high nutritional risk. Goodness of fit was assessed using Hosmer-Lemeshow test. Discrimination ability was assessed based on the area under the receiver operating characteristics curve (AUC). Internal validation was performed using simulated datasets via bootstrapping.

**Results:**

Among the 249 patients, 184 (74%) had moderate to high nutritional risk. Multivariate logistic regression analysis identified stage 3–4 disease (Odds Ratio [OR] 2.54; 95% CI, 1.14–5.69), ECOG performance status of 2–4 (OR 3.04; 95% CI, 1.57–5.88), presence of depression (OR 5.99; 95% CI, 1.99–18.02) and haemoglobin levels <12 g/dL (OR 3.00; 95% CI 1.54–5.84) as significant independent factors associated with moderate to high nutritional risk. The model achieved good calibration (Hosmer-Lemeshow test’s p = 0.17) and discrimination (AUC = 0.80). It retained good calibration and discrimination (bias-corrected AUC = 0.79) under internal validation.

**Conclusion:**

Having advanced stage of cancer, poor performance status, depression and anaemia were found to be predictors of moderate to high nutritional risk. Early identification of patients with these risk factors will allow for nutritional interventions that may improve treatment tolerance, quality of life and survival outcomes.

## Introduction

Malnutrition as defined by the World Health Organisation (WHO) refers to a deficiency of nutrition[1] whilst cachexia is a complex metabolic syndrome associated with underlying cancer and characterized by loss of muscle with or without fat mass[2,3]. It is widely acknowledged that both malnutrition and cachexia are under diagnosed and under treated in patients with cancer[4–6]. Their prevalence varies largely depending on evaluation criteria and has been estimated to range up to as high as 85% in all cancer patients[3,7–10] Malnutrition and cachexia have been shown to be a predictors of risk of toxicity to chemotherapy, impaired quality of life and mortality[3,11–16]. In addition, the experience of weight loss by patients with advanced cancer is distressing for it is viewed as symbolizing proximity of death, loss of control and weakness both emotionally and physically[17].

The elderly patient is particularly prone to inadequate nutritional intake because of factors such as concomitant chronic diseases, polypharmacy, decreased mobility, social changes as well as age related physiological changes[18].

It is a general consensus that malnutrition or cachexia should ideally be recognised in the earlier phase of anti-cancer therapy which offers a window of opportunity for intervention[3]. Early identification of elderly patients at nutritional risk would allow for a quick and timely referral to an appropriately trained professional for a comprehensive nutritional assessment and targeted nutritional intervention which is more likely to be effective before pronounce metabolic deficiencies render them resistant[3].

Evidence based guidelines for the management of elderly patients with cancer recommends a comprehensive geriatric assessment (CGA) to detect unrecognised problems and improve function as well as outcomes[19]. Nutritional assessment is an important component of the CGA. A complete nutritional assessment is complex and usually performed by an appropriately trained professional such as a dietician. An in depth assessment would involve clinical, physical, psychological considerations in addition to anthropometry, biochemical and haematological assessments[8]. This would not be practical for day to day use given the time and manpower constraint of a busy oncology practice.

Nutritional screening on the other hand is quick, easy and provides an indication of a patient’s nutritional risk. Several tools have been designed and available for use in specific patient groups however the absence of a universally agreed criteria in identifying malnutrition has resulted in a lack of consensus among experts as to the “best” or “correct” way of screening for nutritional status [20–23]

One such screening tool is the Nutrition Screening Initiative (NSI) Checklist[20]. The NSI is an American national effort to increase public and health professional awareness of the importance of nutritional problems among older persons[20]. It consists of a self-administered awareness checklist describing characteristics associated with poor nutritional status and was designed to predict adequacy of nutrient intake and overall perceived health[20]. The NSI checklist identifies older persons at nutritional risk due to inadequate nutrient intake as defined by an intake of less than 75% of the recommended daily allowance (RDA) and is used throughout the United States in the assessment of nutrition risk[20].

To date there is a scarcity of studies evaluating malnutrition or nutritional risk in elderly Asian patients. Most of the validated screening tools consist of items such as current weight or body mass index (BMI), decreased dietary intake and unintentional weight loss[23]. None of the available and validated screening tools are based on clinical factors in elderly patients in the setting of a diagnosis of cancer in Asia. We aim to identify CGA based clinical characteristics in elderly Asian cancer patients which are associated with moderate to high nutritional risk as determined by the NSI checklist[20]. These clinical risk factors, which are routinely evaluated in the clinic can form the basis for a simplified screening tool for nutritional risk in the elderly Asian cancer patients.

## Patients and Methods

### Study Design and Patients

This is a retrospective analysis of the CGA data collected from elderly patients attending outpatient oncology clinics at the National Cancer Centre Singapore between May 2007 and November 2010. Patients aged 70 years and older with a diagnosis of cancer at any stage were interviewed by a research nurse prior to their first visit with an oncologist. All patients provided written informed consent before inclusion into the study. The study was approved by the local institutional review board and conducted according to the principles expressed in the Declaration of Helsinki.

### Clinical Data

The CGA questionnaire used in this study was previously described[24] and was developed after a thorough review of the literature and guideline recommendations. The CGA consists of seven distinct domains. Functional status was assessed using Eastern Cooperative Oncology Group (ECOG) performance status[25], the index of activities of daily living (ADL)[26], instrumental activities of daily living (IADL) of Lawton et al[27], the get up and go test[28], and the dominant handgrip strength test. Comorbidities were classified according to the Charlson comorbidity index[29]. Cognitive status was assessed using the mini-mental state examination (MMSE) [30] and clock drawing test[31]. Affective status was assessed via the Geriatric Depression Scale (GDS) Short Form 15[32]. Polypharmacy was documented in terms of number of medications, appropriateness and interactions. Geriatric syndromes were those as described by Balducci et al[33] and nutritional status, was assessed using the body mass index (BMI) and The NSI checklist (Table 1)[20]. The checklist classified patients into low (0 to 2 points), moderate (3 to 5 points) and high nutritional risk (≥6 points) groups. Clinical parameters such as age, sex, stage, tumour types and selected laboratory tests (e.g. haemoglobin, albumin, renal panel, liver function tests) routinely available to treating clinicians were also collected.

**Table 1:** The Nutrition Screening Initiative Checklist.

Statement	Yes
I have an illness or condition that made me change the kind and / or amount of food I eat	2
I eat fewer than 2 meals per day	3
I eat few fruits or vegetables or milk products	2
I have 3 or more drinks of beer, liquor or wine almost everyday	2
I have tooth or mouth problems that make it hard for me to eat	2
I don’t always have enough money to buy the food I need	4
I eat alone most of the time	1
I take 3 or more different prescribed or over-the-counter drugs a day	1
Without want to, I have lost of gained 10 pounds in the last 6 months	2
I am not physically able to shop, cook and / or feed myself	2
Total Score	**/21**

Nutritional score: 0–2 low nutritional risk; 3–5 Moderate nutritional risk, 6 or more High nutritional risk

### Statistical Analysis

Demographic and clinical characteristics between patients with and without moderate to high nutritional risk were compared. Categorical characteristics were compared using the Chi-square test of Fisher’s exact test as appropriate. Mann-Whitney U test was used to compare continuous characteristics between 2 groups of patients. Logistic regression models were fitted to estimate the odds ratios to assess the association of various variables with moderate to high nutritional risk. Considering the large number of significant predictors from the univariate analysis and to avoid model over-fitting, multivariate analyses were performed only on variables with p<0.01 from the univariate analysis. Forward selection, backward elimination and stepwise selection algorithms were applied to identify independent predictors. Goodness of fit between the observed and predicted number of outcomes of the multivariate model were assessed based on the Hosmer-Lemeshow test and its discrimination ability assessed based on the area under the receiver operating characteristics curve (AUC). The AUC was further internally validated based on 200 simulated datasets via bootstrapping to correct for over-fit bias. All p-values were 2 sided and a p-value <0.05 was considered statistically significant. All analyses were performed using SAS version 9.3 (SAS Institute Inc., Cary, NC) and R 2.15.0 (http://www.R-project.org).

## Results

### Patient Characteristics

This analysis included 249 patients with a median age of 77 (range 70–94). Majority of the patients were male (61.4%) and of Chinese race (91.2%). Gastrointestinal (GI) tract cancers were the primary tumor sites in 67.1% of patients followed by lung cancer (11.6%) and genitourinary cancer (4.8%). Most of the patients had late stage cancer (84.7%) and poorer performance status of 2 or greater (66.7%). Table 2 lists the patient characteristics.

**Table 2:** Patient characteristics by nutritional risk.

Variable	Total (n = 249)		Low nutritional risk(n = 65)		Moderate / High nutritional risk (n = 184)		P
	No.	%	No.	%	No.	%	
***Age at CGA assessment*, *years***							
Median (range)	77 (70–94)		76 (70–93)		77 (70–94)		0.903
***Gender***							
Male	153	61.4	45	69.2	108	58.7	0.134
Female	96	38.6	20	30.8	76	41.3	
***Race***							
Chinese	227	91.2	60	92.3	167	90.8	0.073
Malays	12	4.8	2	3.1	10	5.4	
Indians	6	2.4	0	0	6	3.3	
Others	4	1.6	3	4.6	1	0.5	
***Primary tumour site***							
Head and neck	6	2.4	1	1.5	5	2.7	0.003
GI tract	167	67.1	33	50.8	134	72.8	
Breast	5	2.0	1	1.5	4	2.2	
Gynaecologic	2	0.8	2	3.1	0	0	
Lung	29	11.6	15	23.1	14	7.6	
Lymphoma	2	0.8	0	0	2	1.1	
Genitourinary	12	4.8	4	6.2	8	4.3	
Dual primaries	7	2.8	1	1.5	6	3.3	
Others	19	7.6	8	12.3	11	6.0	
***Stage at diagnosis***							
Early (I–II)	38	15.3	19	29.2	19	10.4	<0.001
Late (III–IV)	210	84.7	46	70.8	164	89.6	
***ECOG performance status***							
0–1	83	33.3	38	58.5	45	24.5	<0.001
2–4	166	66.7	27	41.5	139	75.5	
***Activities of daily living***							
Independent (A–F)	204	81.9	62	95.4	142	77.2	0.001
Dependent (G & Others)	45	18.1	3	4.6	42	22.8	
***Instrumental activities of daily living***							
< 7	219	88.3	49	76.6	170	92.4	0.001
≥ 7	29	11.7	15	23.4	14	7.6	
***Get up and go test***							
Normal	81	32.8	28	43.1	53	29.1	0.011
Very slightly abnormal	80	32.4	25	38.5	55	30.2	
Mildly abnormal	35	14.2	8	12.3	27	14.8	
Moderately abnormal	19	7.7	2	3.1	17	9.3	
Severely abnormal	32	13.0	2	3.1	30	16.5	
***Dominant handgrip strength test*, *kg***							
Median (range)	30 (0–90)		40 (3.3–90)		26.7 (0–80)		<0.001
***Charlson comorbidity index***							
Low	83	33.3	22	33.8	61	33.2	0.845
Medium	116	46.6	31	47.7	85	46.2	
High	37	14.9	10	15.4	27	14.7	
Very high	13	5.2	2	3.1	11	6.0	
***Clock drawing test score***							
Normal (≤2)	96	41.7	32	51.6	64	38.1	0.065
Abnormal (>2)	134	58.3	30	48.4	104	61.9	
***Mini-mental state examination score***							
Normal (≥24)	163	67.6	53	82.8	110	62.1	0.003
Abnormal (<24)	78	32.4	11	17.2	67	37.9	
***Geriatric depression scale***							
Normal (≤5)	177	71.7	60	93.8	117	63.9	<0.001
Depressed (>5)	70	28.3	4	6.3	66	36.1	
***Caregiver burden***							
Little or no burden	188	77.0	57	89.1	131	72.8	0.013
Mild to moderate burden	55	22.5	7	10.9	48	26.7	
Moderate to severe burden	1	0.4	0	0	1	0.6	
***Polypharmacy (>4 prescribed drugs)***							
No	98	39.5	36	56.3	62	33.7	0.002
Yes	150	60.5	28	43.8	122	66.3	
***Presence of geriatric syndromes***							
No	98	39.4	41	63.1	57	31.0	<0.001
Yes	151	60.6	24	36.9	127	69.0	
***BMI***							
< 27.5	232	93.5	55	85.9	177	96.2	0.007
≥ 27.5	16	6.5	9	14.1	7	3.8	
***Haemoglobin*, *g/dL***							
Normal (≥12)	106	43.3	43	68.3	63	34.6	< 0.001
Abnormal (<12)	139	56.7	20	31.7	119	65.4	
***Creatinine clearance test*, *ml/min***							
Normal (≥60)	156	68.1	40	71.4	116	67.1	0.541
Abnormal (<60)	73	31.9	16	28.6	57	32.9	
***Albumin*, *g/L***							
Normal (>35)	53	22.9	25	42.4	28	16.3	< 0.001
Abnormal (≤35)	178	77.1	34	57.6	144	83.7	
***Bilirubin*, *μmol/L***							
Normal (≤24)	195	84.8	49	84.5	146	84.9	0.941
Abnormal (>24)	35	15.2	9	15.5	26	15.1	
***ALT*, *U/L***							
Normal (≤36)	186	80.5	49	84.5	137	79.2	0.379
Abnormal (>36)	45	19.5	9	15.5	36	20.8	
***AST*, *U/L***							
Normal (≤33)	145	63.0	37	64.9	108	62.4	0.736
Abnormal (>33)	85	37.0	20	35.1	65	37.6	

Abbreviations: CGA, comprehensive geriatric assessment; ECOG, Eastern Coorperative Oncology Group; BMI, Body Mass Index; ALT, alanine transaminase; AST, aspartate transaminase

### Patient characteristics by nutritional risk

A significant proportion of patients (73.9%) were at moderate to high nutritional risk. Compared with patients with low nutritional risk, there were significantly more patients with moderate to high nutritional risk who had primary tumour in the GI tract (73% vs 51%), ECOG performance status 2–4 (76% vs 42%), advanced stage of disease at diagnosis (90% vs 71%), depression based on geriatric depression scale (36% vs 6%), low MMSE scores (< 24 points) (38% vs 17%), imposed mild to severe burden to their caregivers (27% vs 11%), had more than 4 prescribed drugs (66% vs 44%) and the presence of geriatric syndromes (69% vs 37%) (all p < 0.02).

Patients with moderate to high nutritional risk also had significantly lower median BMI values (20.9 vs 23.7), haemoglobin levels (11.1 vs 12.5 g/dL), and albumin levels (29.0 vs 34.0 g/L) (all p < 0.001).

There were no significant differences in age, gender, comorbidity risk, renal and liver functions between the 2 groups of patients.

### Univariate logistic regression analysis

Factors that were significantly associated with moderate to high nutritional risk included an advanced stage at diagnosis [odds ratio (OR) 3.57; 95% confidence interval (CI) 1.74–7.29], a higher ECOG performance status of 2–4 (OR 4.35; 95% CI 2.39–7.90), being dependent in ADL (OR 6.11; 95% CI 1.83–20.47), a lower score in IADL (OR 1.43; 95% CI 1.23–1.67), a lower score in dominant handgrip strength test (OR 0.95; 95% CI 0.94–0.97), MMSE score < 24 (OR 2.94; 95% CI 1.43–6.01), GDS score > 5 (OR 8.46; 95% CI 2.94–24.33), presence of geriatric syndromes (OR 3.81; 95% CI 2.10–6.89), imposing mild to severe burden to caregivers (OR 3.05; 95% CI 1.30–7.13), having more than 4 prescribed drugs (OR 2.53; 95% CI 1.42–4.52), a lower BMI value (OR 1.23; 95% CI 1.14–1.35), lower haemoglobin levels (OR 1.43; 95% CI 1.22–1.69) and lower albumin levels (OR 1.14; 95% CI 1.08–1.20) (Table 3).

**Table 3:** Univariate logistic regression of moderate to high nutritional risk.

Variable	Categories	OR	95% CI	*P*
Primary tumour site	GI tract vs Head & neck	0.81	0.09–7.19	0.044
	Breast vs Head & neck	0.80	0.04–17.20	
	Gynaecologic vs Head & neck	NE	NE	
	Lung vs Head & neck	0.19	0.02–1.80	
	Lymphoma vs Head & neck	NE	NE	
	Genitourinary vs Head & neck	0.40	0.03–4.68	
	Dual primaries vs Head & neck	1.20	0.06–24.47	
	Others vs Head & neck	0.28	0.03–2.83	
Stage at diagnosis	Late (III–IV) vs Early (I–II)	3.57	1.74–7.29	0.001
Metastasis at diagnosis	Yes vs No	1.79	1.01–3.17	0.048
ECOG performance status	2–4 vs 0–1	4.35	2.39–7.90	<0.001
ADL	Dependent (G & Others) vs Independent (A–F)	6.11	1.83–20.47	0.003
Instrumental ADL	≥ 7 vs < 7	0.27	0.12–0.60	0.001
Get up and go test	Very slightly abnormal vs Normal	1.16	0.60–2.25	0.027
	Mildly abnormal vs Normal	1.78	0.72–4.44	
	Moderately abnormal vs Normal	4.49	0.97–20.84	
	Severely abnormal vs Normal	7.92	1.76–35.61	
Dominant handgrip strength test	Per kg increase	0.95	0.94–0.97	<0.001
Clock drawing test score	Abnormal (>2) vs Normal (≤2)	1.73	0.96–3.12	0.067
Mini-mental state examination score	Abnormal (<24) vs Normal (≥24)	2.94	1.43–6.01	0.003
Geriatric depression scale	Depressed (>5) vs Normal (≤5)	8.46	2.94–24.33	<0.001
Caregiver burden	Mild to severe vs Little or no	3.05	1.30–7.13	0.010
Polypharmacy	Yes vs No	2.53	1.42–4.52	0.002
BMI	≥ 27.5 vs < 27.5	0.24	0.09–0.68	0.007
Haemoglobin, g/dL	Abnormal (<12) vs Normal (≥12)	4.06	2.20–7.49	<0.001
Albumin, g/L	Abnormal (≤35) vs Normal (>35)	3.78	1.96–7.29	<0.001
Geriatric syndromes	Yes vs No	3.81	2.10–6.89	<0.001

Abbreviations: OR, odds ratio; CI, confidence interval; NE, not estimable; ECOG, Eastern Cooperative Oncology Group; ADL, activities of daily living; BMI, body mass index

### Multivariate logistic regression analysis

Multivariate logistic regression analysis using forward selection, backward elimination and stepwise selection algorithms identified identical predictors for moderate to high nutritional risk (Table 4). Stage 3–4 at diagnosis (OR 2.54; 95% CI 1.14–5.69; p = 0.023), ECOG performance status of 2–4 (OR 3.04; 95% CI 1.57–5.88; p = 0.001), presence of depression as measured by GDS (OR 5.99; 95% CI 1.99–18.02; p = 0.001) and haemoglobin levels < 12 g/dl (OR 3.00; 95% CI 1,54–5.84; p = 0.001) were all statistically significant independent factors associated with moderate to high nutritional risk.

**Table 4:** Multivariate logistic regression of moderate to high nutritional risk.

Variable	Categories	OR	95% CI	*P*
Stage at diagnosis	Late (III–IV) vs Early (I–II)	2.54	1.14–5.69	0.023
ECOG performance status	2–4 vs 0–1	3.04	1.57–5.88	0.001
Geriatric depression scale	Depressed (>5) vs Normal (≤5)	5.99	1.99–18.02	0.001
Haemoglobin, g/dL	Abnormal (<12) vs Normal (≥12)	3.00	1.54–5.84	0.001

Abbreviation: OR, odds ratio; CI, confidence interval; ECOG, Eastern Cooperative Oncology Group

### Clinical scoring system

**Fig 1:**
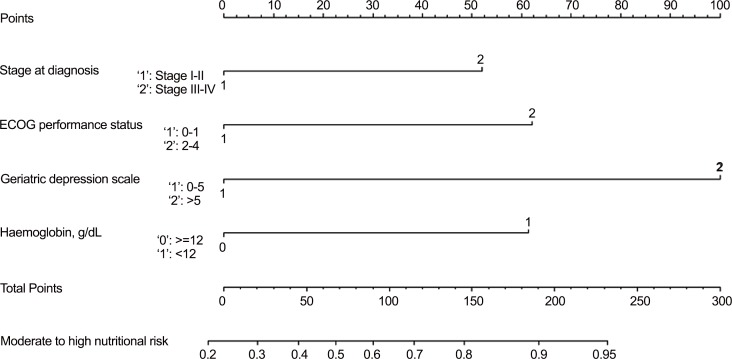
Nomogram for moderate to high nutritional risk in an elderly Asian cancer patient. The predicted probability of moderate to high nutritional risk of a patient is obtained by first locating the patient’s stage at diagnosis, Eastern Cooperative Oncology Group [ECOG] performance status, geriatric depression scale and haemoglobin on each axis. Draw a vertical line to the “points” axis to determine the number of points to assign for each variable’s value. Sum all the points for all variables, locate the total sum on the “Total Points,” and draw a straight line down to locate the probability of moderate to high nutritional risk corresponding to the sum.

**Fig 2:**
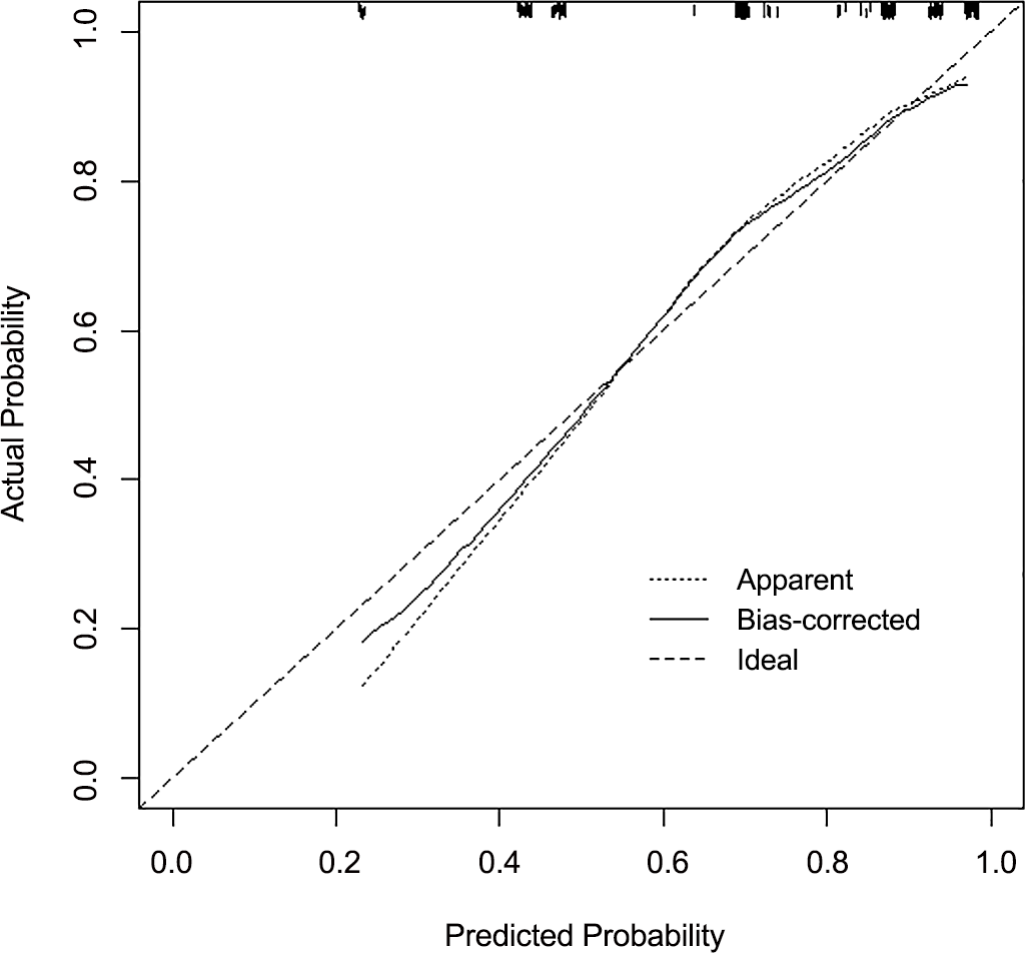
Calibration plot of the final model for moderate to high nutritional risk.

A nomogram was constructed based on the multivariate model as shown in Fig 1. The model achieved both calibration (Hosmer-Lemeshow test’s p = 0.172) and discrimination (AUC = 0.799). Based on bootstrapping, the bias-corrected AUC of the multivariate model was slightly lower at 0.788, indicating that the model retained a good discrimination. The predicted probabilities of moderate to high nutritional risk based on the model approximated the actual outcomes well (Fig 2).

## Discussion

We have previously reported nutritional risk as assessed using the NSI to be predictive of survival in elderly Asian patients with cancer[24]. We have shown here a high prevalence (73.9%) of nutritional risk in our cohort of elderly Asian cancer patients. To our knowledge, our study is the first to investigate the relationship between nutritional risk, defined by the NSI and all domains of the CGA in addition to readily available clinical parameters specifically in a cohort of elderly Asian patients with cancer. We have identified four factors; presence of depression, advanced stage, poor performance status, and anaemia as significantly associated on multivariate analysis with moderate to high nutritional risk.

In a recent cohort study (The ELCAPA-05), the Mini Nutritional Assessment (MNA) was used as the primary evaluation criterion[7]. This tool requires a professional to complete and evaluates risk of undernutrition through measures of anthropometry, dietary and clinical global assessment in addition to self-perception of health and nutritional status[34]. A total of 643 patients were included in the survey[7]. Similar to our study, the authors highlighted a high prevalence of malnutrition of 20.7% and 43.5% at risk of malnutrition in their cohort of elderly French cancer patients[7]. The presence of geriatric syndromes such as cognitive impairment, depressed mood and fall risk were independent risk factors for malnutrition[7]. In particular, depressed mood was associated with a 1.5–3 times risk for malnutrition in their cohort of patients[7]. The relationship between nutritional status and psychological status in patients with colorectal cancers was investigated in a Canadian study not limited to elderly patients[35]. Depression was identified as an independent predictor of risk of malnutrition when controlling for age, gender, marital status and weight change[35]. Further work is required to investigate the causal relationship between depression and malnutrition.

Advanced tumor stage, a consequence of disease progression is a well-established poor prognostic factor[14,24]. Nutritional risk likely reflects the consequence of having advanced disease and the general health of patients. In a large study of 14972 Korean cancer patients, the proportion of patients with high risk for malnutrition as defined by BMI, serum albumin, total lymphocyte count and dietary intake, increased with cancer stage[36]. Similarly, in the SCReening the Nutritional status in Oncology (SCRINIO) study of 1000 oncology outpatients in Italy, weight loss was higher in patients with more advanced stage of disease and compromised performance status[37].

Nutritional risk as defined by the Nutritional Risk Score (NRS) was noted to be higher in patients with poorer performance status[37]. Similarly, a multicenter observational study conducted in France identified a WHO performance status score of 2 or more as a risk factor for malnutrition as defined by 2 anthropometric indicators, the level of weight loss and BMI[15]. Performance status is a commonly cited factor independently associated with mortality[38].

Anaemia, a common finding in patients with cancer may adversely influence the management of elderly cancer patients by limiting dose intensity of treatment and hence affecting efficacy. In a prospective survey, Mancuso et al analyzed the correlation between CGA parameters and anemia[39]. Functional decline, cognitive decline, depression and poor quality of life were identified as associated with low haemoglobin levels[39]. In a review of the literature of elderly cancer patients, anaemia has not yet been found to be a predictor for risk of malnutrition. Hence this is the first study to report this association.

Early identification of malnutrition allows for timely referral to appropriately trained health care professionals leading to interventions that may modify risk factors and potentially improve outcomes. We report here an exploratory analysis identifying four factors that should be further explored for subsequent use in clinical trials and therapeutic recommendations. We have incorporated these four factors in developing a clinical scoring system to predict an individual elderly patient’s risk for malnutrition. As far as we are aware, this is the first scoring system utilizing clinical factors and parameters providing an individualised malnutrition risk assessment in this unique population of patients.

There are however some limitations to our study. The NSI checklist was originally applied in a cohort of non-instituitionalised, white, older persons without a specific diagnosis of cancer[20]. Few studies have validated the NSI checklist and data for its predictive value with regards to mortality remains weak[18,40–42]. Our study population is small and heterogeneous in terms of the tumor types. The patients included in our analysis are outpatients representing a group of fitter patients. The majority of our patients had GI tract cancers with an underrepresentation of other solid tumour types. This reflects selection bias in the conduct of this study the results of which may therefore not be completely extrapolated to the general elderly cancer patient population. GI tract cancer patients may have higher risk of malnutrition due to the site and nature of their disease compared to those with other tumor types. Given several reports on varying prevalence of malnutrition based on primary tumour sites[36], future studies should be conducted focusing on specific tumor types.

In taking the findings of this study to the next step, we plan to prospectively validate this score in a separate population of elderly Asian cancer patients.

In conclusion, a significant number of elderly Asian cancer patients are at nutritional risk. Physicians need to have a strong index of suspicion of under nutrition in the elderly population. Advanced stage of cancer, poor performance status, depression and anaemia are independent predictors of moderate to high nutritional risk.
